# Ultrasound‐guided hookwire localization of non palpable cervical lymphadenopathy: A case–control study of operative time

**DOI:** 10.1002/cam4.6257

**Published:** 2023-06-14

**Authors:** William Bran, Sonia Sahli‐Vivicorsi, Romain Cadieu, Zarrin Alavi, Jean‐Christophe Leclere

**Affiliations:** ^1^ Radiology Department Brest University Hospital Brest France; ^2^ ENT Department Brest University Hospital Brest France; ^3^ INSERM, CIC 1412 Brest University Hospital Brest France

**Keywords:** cervical lymph node, cervical lymphadenopathy, hookwire‐guided localization, nonpalpable, operative time, recurrent nodal disease, ultrasound

## Abstract

**Objective:**

We aimed at evaluating the impact of ultrasound‐guided (US) hookwire localization of nonpalpable cervical lymphadenopathy on operating time.

**Design and Methods:**

Retrospective case control study (January 2017 and May 2021) of 26 patients with lateral nonpalpable cervical lymphadenopathy undergoing surgery with (H+) and without (H−) per operative US‐guided hook‐wire localization. Operative time (general anesthesiology onset, hookwire placement, end of surgery) and surgery‐related adverse events data were collected.

**Results:**

Mean operative time was significantly shorter in H+ group versus H− group (26 ± 16 min vs. 43 ± 22 min) (*p* = 0.02). Histopathological diagnosis accuracy was 100% versus 94% (H+ vs. H−, *p* = 0.1). No significant between group difference in surgery‐related adverse events was reported (wound healing, *p* = 0.162; hematomas, *p* = 0.498; neoplasms removal failure, *p* = 1).

**Conclusion:**

US‐guided hookwire localization of lateral nonpalpable cervical lymphadenopathy allowed a significant reduction in operative time, comparable histopathological diagnosis accuracy and adverse events compared with H−.

## Graphical abstract

Per operative ultrasound‐guided hookwire localization versus nonhookwire localization of nonpalpable cervical lymphadenopathy allowed a significant reduction in operative time, comparable histopathological diagnosis accuracy and adverse events.

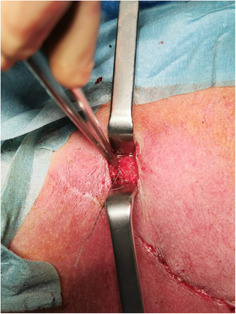

## 1 INTRODUCTION

Screening for suspicion of sub‐clinical cervical adenopathy using imaging tools such as PET‐CT has led to high diagnosis accuracy, that is, with 80% sensitivity^1^, ^2^ and 100% specificity when the SUV‐max is greater than 5.5.^3^ Such detection tools should be combined with histological evidence to achieve more accurate and reliable diagnosis and thus most optimal treatment management. Given the absence or lack of sufficient number of malignant cells, ultrasound‐guided (US‐guided) cytopuncture, widely used postradiotherapy, has an estimated nondiagnostic yield of 10%–30%.^4^, ^5^, ^6^, ^7^, ^8^ Further investigation such as adenectomy is required for positive diagnosis and treatment management.

Surgical removal of adenopathy for histological or microbiological purposes is a common procedure in cervical surgery. The latter can lead to adverse events such as vascular and lymphatic wounds and nerve damage (mandibular branch of VII, X, XI, and XII, sympathetic chain, brachial plexus, phrenic and lingual nerve).^9^, ^10^ Neck surgery exploration is more difficult in cases of nonpalpable lesions. Other factors such as postsurgery and postradiotherapy tissue damages can also increase adverse events^11^ and false negatives rates.

Preoperative noninvasive imaging workup (e.g., PET‐CT, CT, US, MR) can provide helpful data on the lesion and its environment–yet it does not provide perioperative imaging. Perioperative US‐imaging sets forth the lag time imaging discrepancies between the initial workup and the surgery, that is, discrepancies according to patient and clinical factors (e.g., patient position and treatments).

Several techniques have been used for localization of nonpalpable cervical lesions, mainly in recurrent thyroid cancer,^12^, ^13^, ^14^, ^15^ aiming at reducing nerve damage.^16^

Hookwire localization of a suspicious nonpalpable lesion prior to surgical removal is routinely used in the treatment of breast cancer.^17^, ^18^, ^19^, ^20^ This helps not only at providing a diagnostic decision for all imaging‐screened suspicious nodes but also at reducing the risk of damage to the neighboring unaffected tissues.^21^ Hookwire localization has also been described in orthopedic surgery^22^, ^23^, ^24^ and thoracic surgery.^25^, ^26^

The use of ultrasound or CT‐guided hookwire in cervical surgery was recently reviewed^27^, ^28^ and several studies reported its use in resection of benign or malignant lesions.^11^, ^29^, ^30^, ^31^, ^32^, ^33^, ^34^, ^35^, ^36^, ^37^, ^38^, ^39^, ^40^, ^41^, ^42^ There are only four reported cases of lateral cervical adenectomy using US‐guided hookwire localization.^37^, ^38^, ^39^ Park et al. reported a series of eight cases of preoperative ultrasound‐guided hookwire needle localization of nonpalpable cervical mass.^40^ To our knowledge, the impact of the use of the ultrasound‐guided hookwire on the operative time has not been evaluated in the literature.

The primary objective of our study was to compare the operative time of cervical adenectomies with (H+) and without (H−) intraoperative ultrasound‐guided hookwire localization. The secondary objectives were to compare diagnostic performance and adverse events rates.

## 2 MATERIALS AND METHODS

This is a single‐center retrospective case control study conducted at the Brest University Hospital between January 2017 and May 2021. This study was written following the STROBES recommendations for observational studies and was approved by the ethics committee (IRB of Brest University Hospital).

### 2.1 Patient selection

**FIGURE 1:**
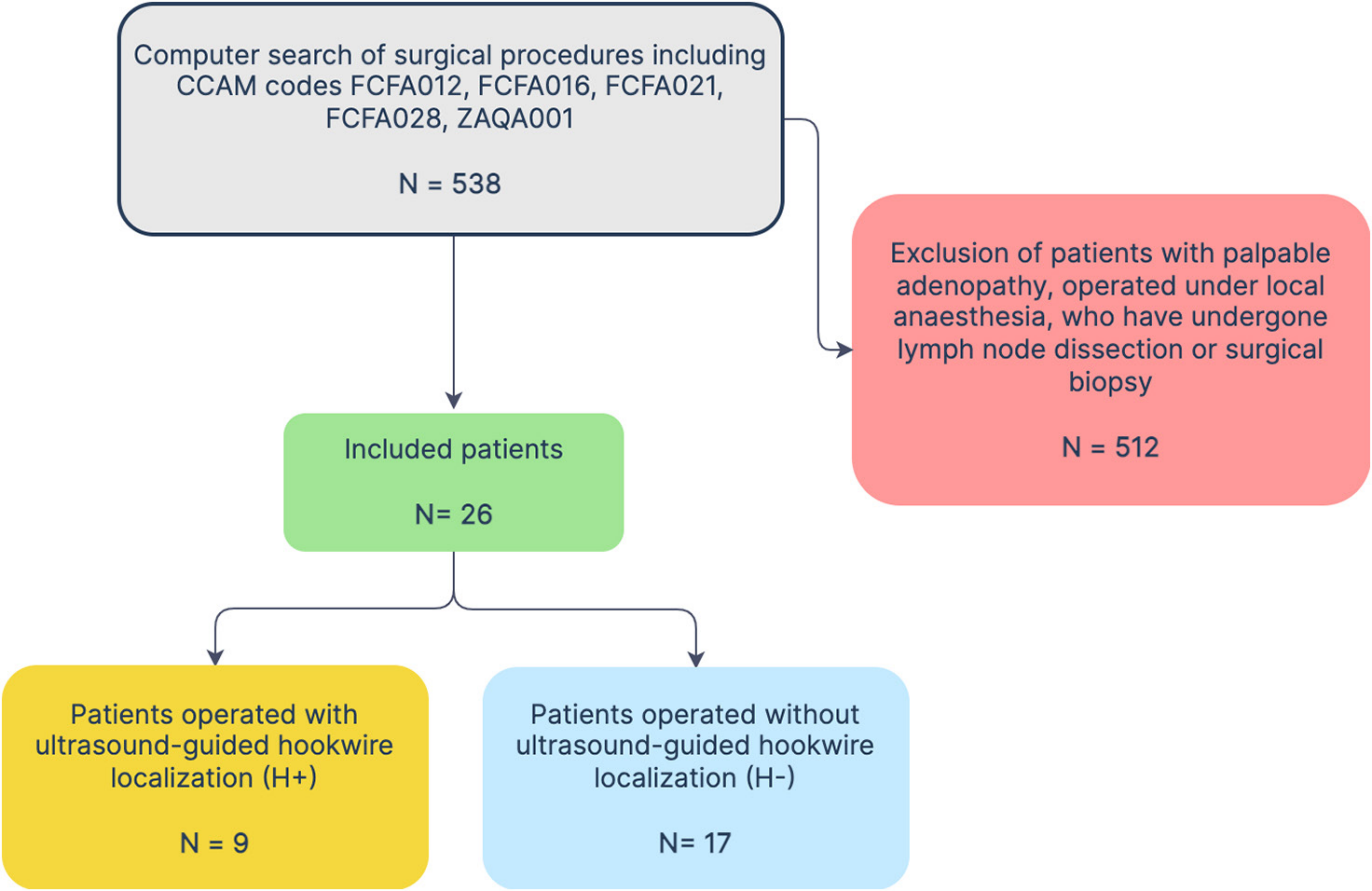
Patient selection. FCFA012: diagnostic neck lymph node removal by cervicotomy; FCFA016: partial unilateral cervical lymph node removal by cervicotomy; FCFA021: diagnostic limb lymph node removal by direct approach; FCFA028: therapeutic neck lymph node removal by cervicotomy; ZAQA001: neck exploration by cervicotomy.

Inclusion and exclusion criteria are reported in the Figure 1.

### 2.2 Group with ultrasound‐guided hookwire localization (H+)

H+ group included all patients with nonpalpable adenopathy on clinical examination who underwent adenoectomy under GA. For eight patients, the suspicious cervical adenopathy was detected by PET‐CT, for one patient it was detected by US. A total of 13 adenectomy with ultrasound‐guided hookwire localization were performed in nine patients (H+ group). The general characteristics of the patients are displayed in Table 1.

**TABLE 1:** General characteristics of patients operated with ultrasound‐guided hookwire localization (H+).

Patient	Age (years), sex	BMI	Initial cancer	History of neck surgery	History of cervical radiotherapy	Number and location of adenopathy	Cytopuncture and result	Histology	Number of hookwire (*n*)
1	70, M	28	Lingual SCC	Yes	Yes	2 IA	Yes, −	SCC	2
2	51, M	25	PTC	Yes	No	1 IV	Yes, +	PTC	1
3	57, M	18	Tonsil SCC	No	Yes	1 IIA	Yes, +	SCC	1
4	78, F	23	Buccal SCC	Yes	Yes	1 IB	Yes, +	SCC	1
5	52, M	25	CUP SCC	Yes	No	3 IIA, IIB, III	No	SCC × 2 Benin	4
6	55, M	24	Absence	No	No	2 IIB	Yes, −	Benin	1
7	66, M	33	Tonsil SCC	No	Yes	1 IV	No	Necrosis	1
8	74, F	27	Retromolar trigone SCC	Yes	Yes	1 IB	Yes, +	Fibrosis	1
9	62, F	25	Absence	No	No	1 IV	Yes, −	Benin	1

Abbreviations: CUP, cancer of unknown primary; F, female; M, male; PTC, papillary thyroid carcinoma; SCC, squamous cell carcinoma.

### 2.3 Group without ultrasound‐guided hookwire localization (H−)

All patients had nonpalpable adenopathy on clinical examination. For 16 patients, the suspicious cervical adenopathy was detected by PET‐CT, for one patient it was detected by CT. General characteristics of the patients are displayed in Table 2.

**TABLE 2:** General characteristics of H− patients.

Patient	Age (years), sex	BMI	Initial cancer	History of neck surgery	History of cervical radiotherapy	Number and location of adenopathy	Cytopuncture	Histology
1	62, F	31	Absence	No	No	1 IV	No	Sarcoidosis
2	81, M	30	Absence	No	No	1 IV	Yes, −	Nonpathological
3	80, F	24	Ovarian adenocarcinoma	Yes	No	1 IV	Yes, +	Ovarian adenocarcinoma
4	48, M	27	Absence	Yes	No	1 III	No	Lymphoma
5	47, M	26	Absence	No	No	1 IIA, III	No	Lymphoma
6	80, M	32	Lip adenocarcinoma	Yes	Yes	1 IB	Yes, −	SCC
7	59, M	21	Tonsil SCC	Yes	No	1 IIA	Yes +	SCC
8	69, F	38	Breast	No	No	1 IB, IIA	No	Breast carcinoma + lymphoma
9	61, M	25	Cutaneous SCC	No	Yes	1 IB	Yes, −	SCC
10	29, F	22	PTC	Yes	No	1 IIA	Yes, −	PTC
11	76, M	22	Absence	No	No	1 IV	Yes, −	Lymphoma
12	70, M	33	Absence	No	No	1 III	Yes, −	Sarcoidosis
13	86, F	26	Absence	No	No	1 V	Yes, −	Inflammatory
14	75, F	22	Submandibular gland ACC	Yes	Yes	1 IIA	No	Failed surgery
15	64, M	35	Hypopharynx SCC	No	Yes	1 IIA	No	Nonpathological
16	61, M	20	Tongue SCC	No	Yes	1 IIB	No	SCC
17	52, M	33	Renal cell carcinoma	No	No	1 IIA	No	Renal cell carcinoma

Abbreviations: ACC, adenoid cystic carcinoma; BMI, body mass index; F, female; M, male; PTC, papillary thyroid carcinoma; SCC, squamous cell carcinoma.

### 2.4 Ultrasound‐guided hookwire localization technique

**FIGURE 2:**
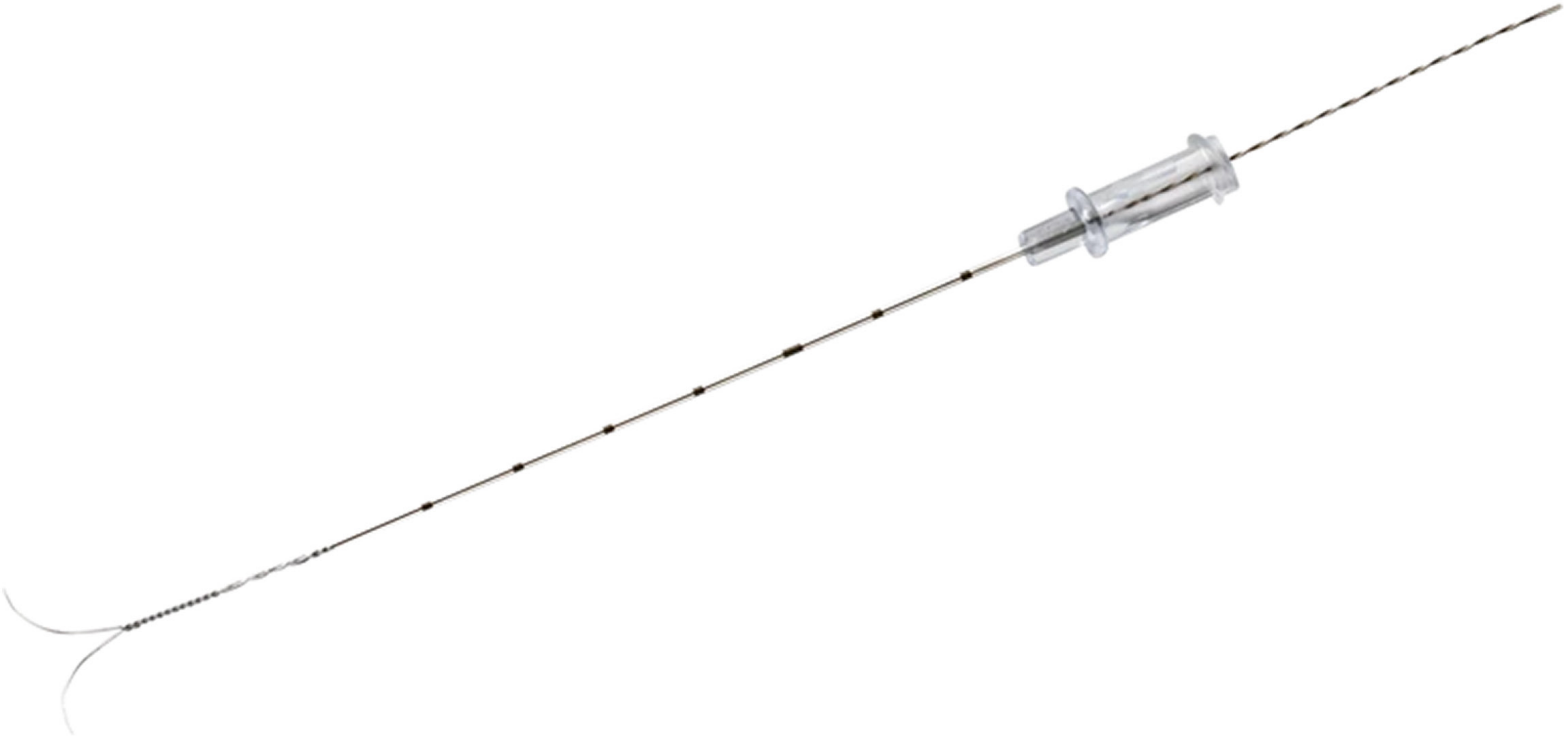
DUALOK hookwire, Bard France SA, used in our study.

All hook‐wires were placed in the operating room after GA onset by the same radiologist specialized in ENT and breast imaging. After general anesthesia and orotracheal intubation, patients were placed in supine position, with the head turned contralateral to the adenopathies, slightly hyperextended. The real‐time US‐guided localization of the suspicious lesion was done using a Samsung UGEO‐HM70A (Samsung Medison Co. Ltd) ultrasound machine at a high frequency linear probe (14 MHz) protected by a sterile sheath. A 20G (0.81 mm) hook‐wire (Dualok, Bard France SA, reference LW) was introduced under ultrasound guidance (Figure 2).

**FIGURE 3:**
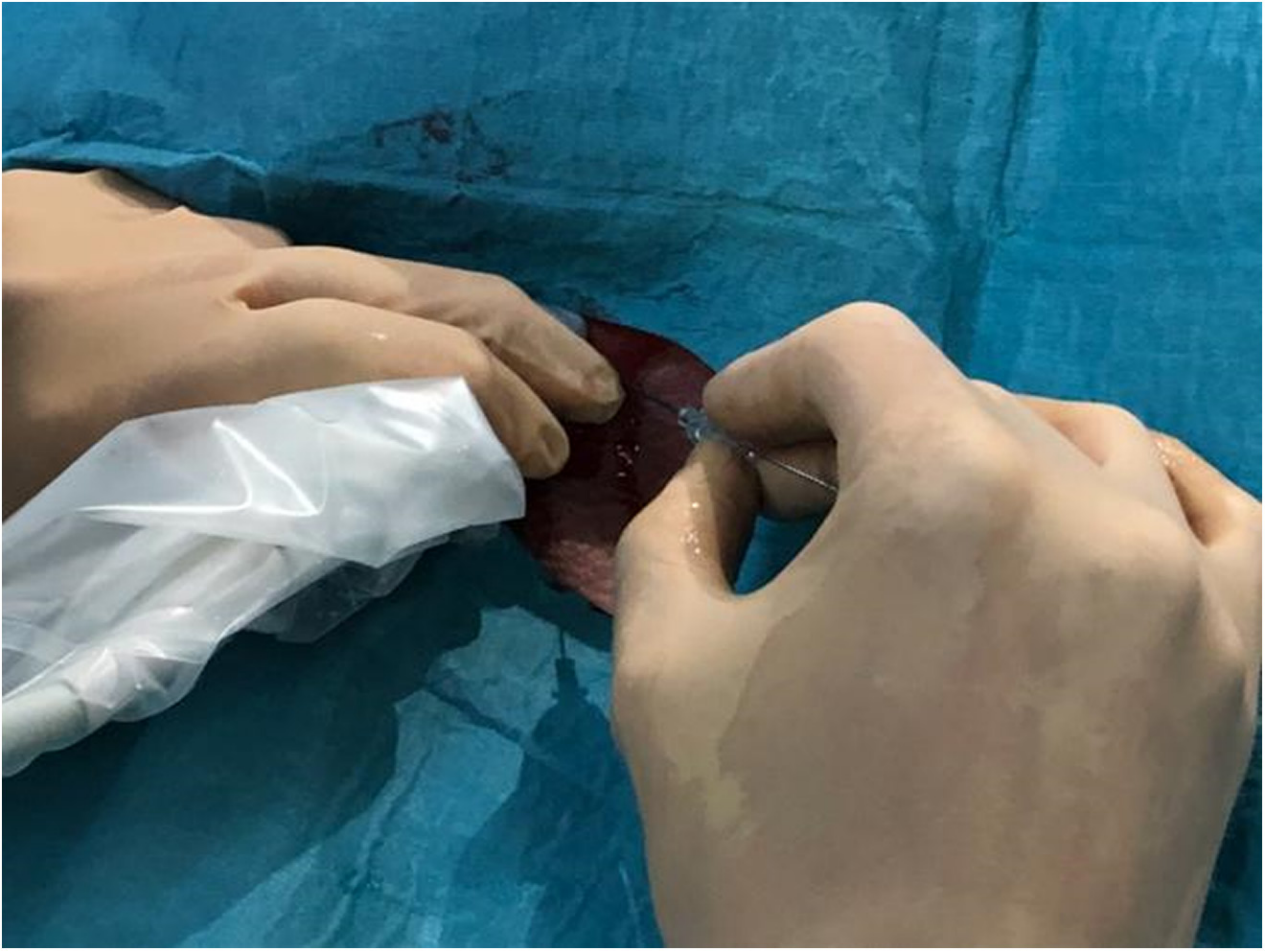
Introduction of the hook‐wire under ultrasound.

**FIGURE 4:**
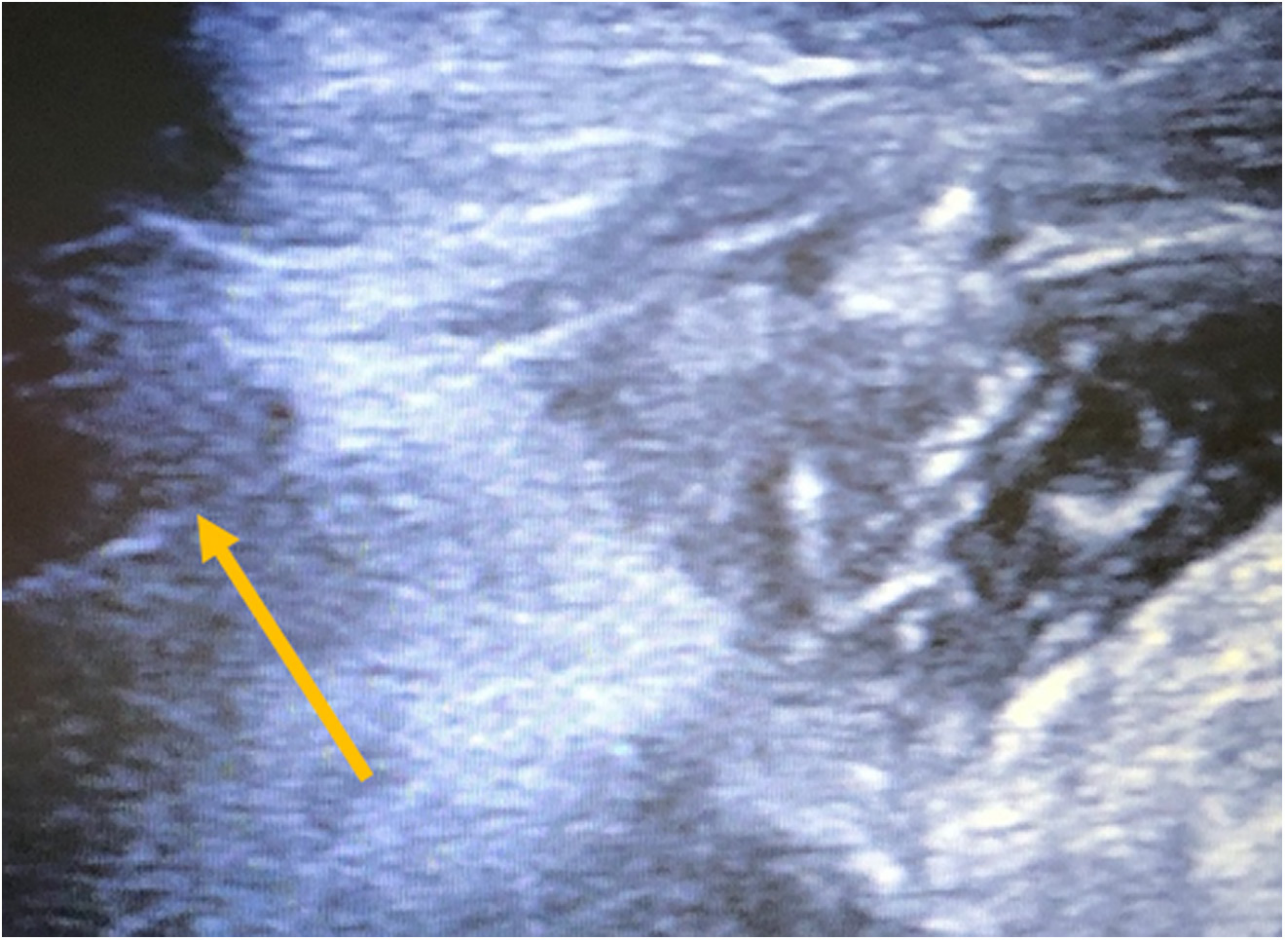
Hook‐wire placement under ultrasound yellow arrow shows the hook‐wire guide inside the suspicious lesion).

**FIGURE 5:**
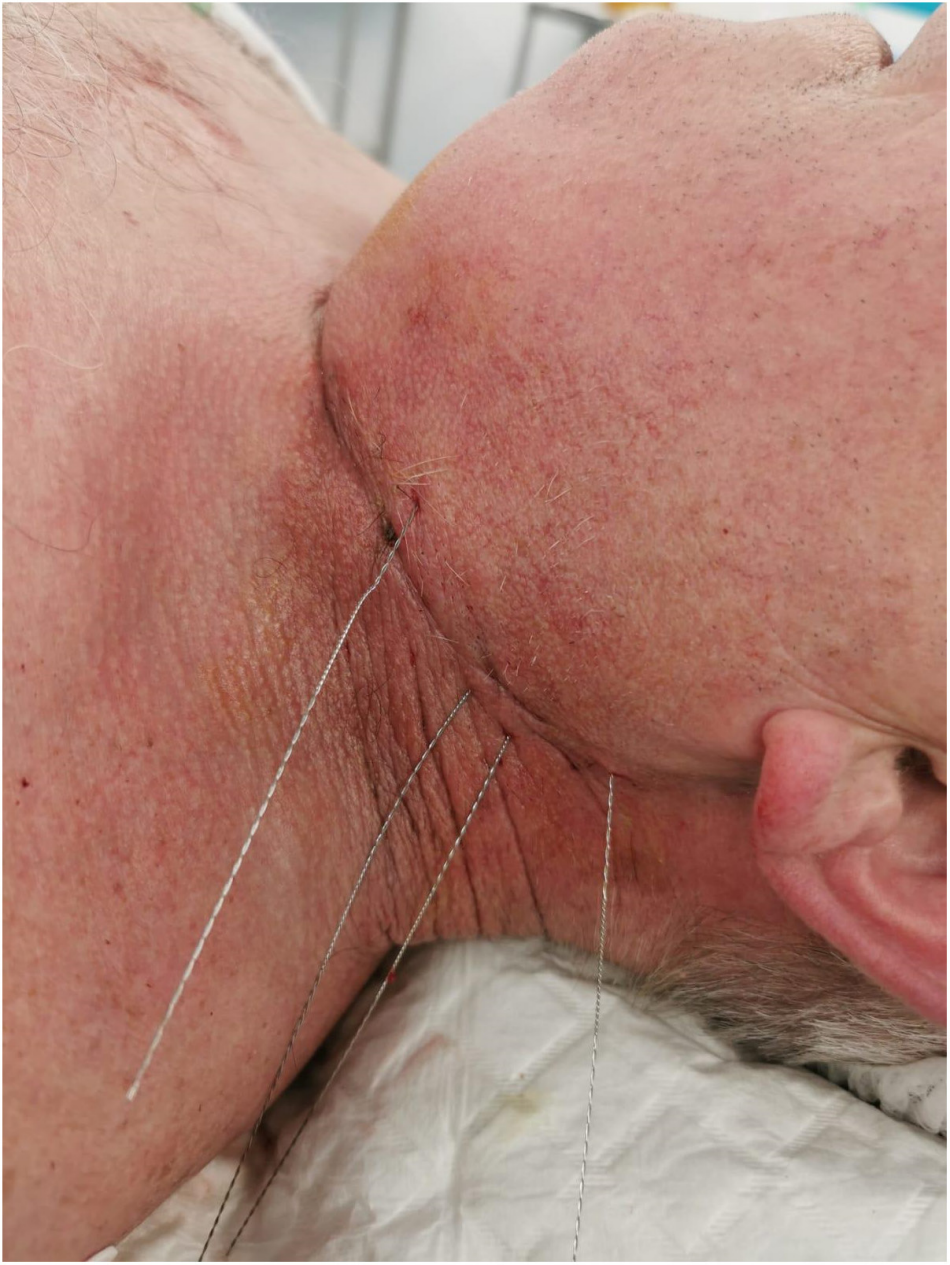
Four Hook‐wires in place in three adenopathies intraoperatively before surgical removal.

The hook‐wire placement (i.e., inside or adjacent to the target) using the perpendicular approach was done to achieve the shortest possible distance between the skin penetration site and the target. This was done taking into account the size, depth and proximity of the lesion to adjacent vascular structures (Figures 3 and 4). A consensus for hook‐wire placement was reached after discussion of all options between the surgeon and the radiologist. The guiding needle was removed and the wire was left onto the skin. Several hook‐wires could be placed at the same time in case of suspicion of more than one lesion (Figure 5). The surgeon was informed of the depth and position of the hook‐wire.

### 2.5 Surgical technique and histological analysis

**FIGURE 6:**
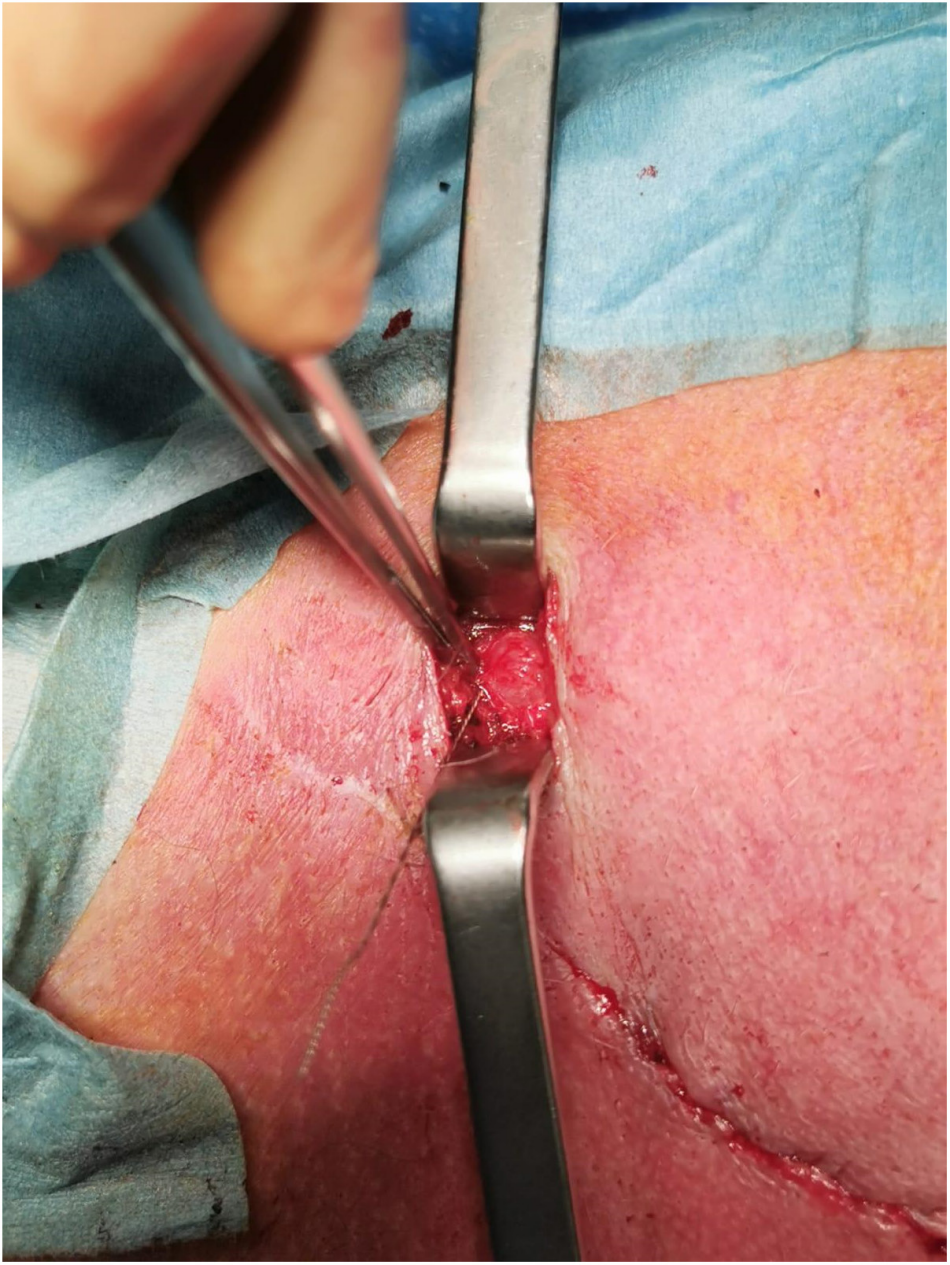
Hook‐wire‐guided dissection.

**FIGURE 7:**
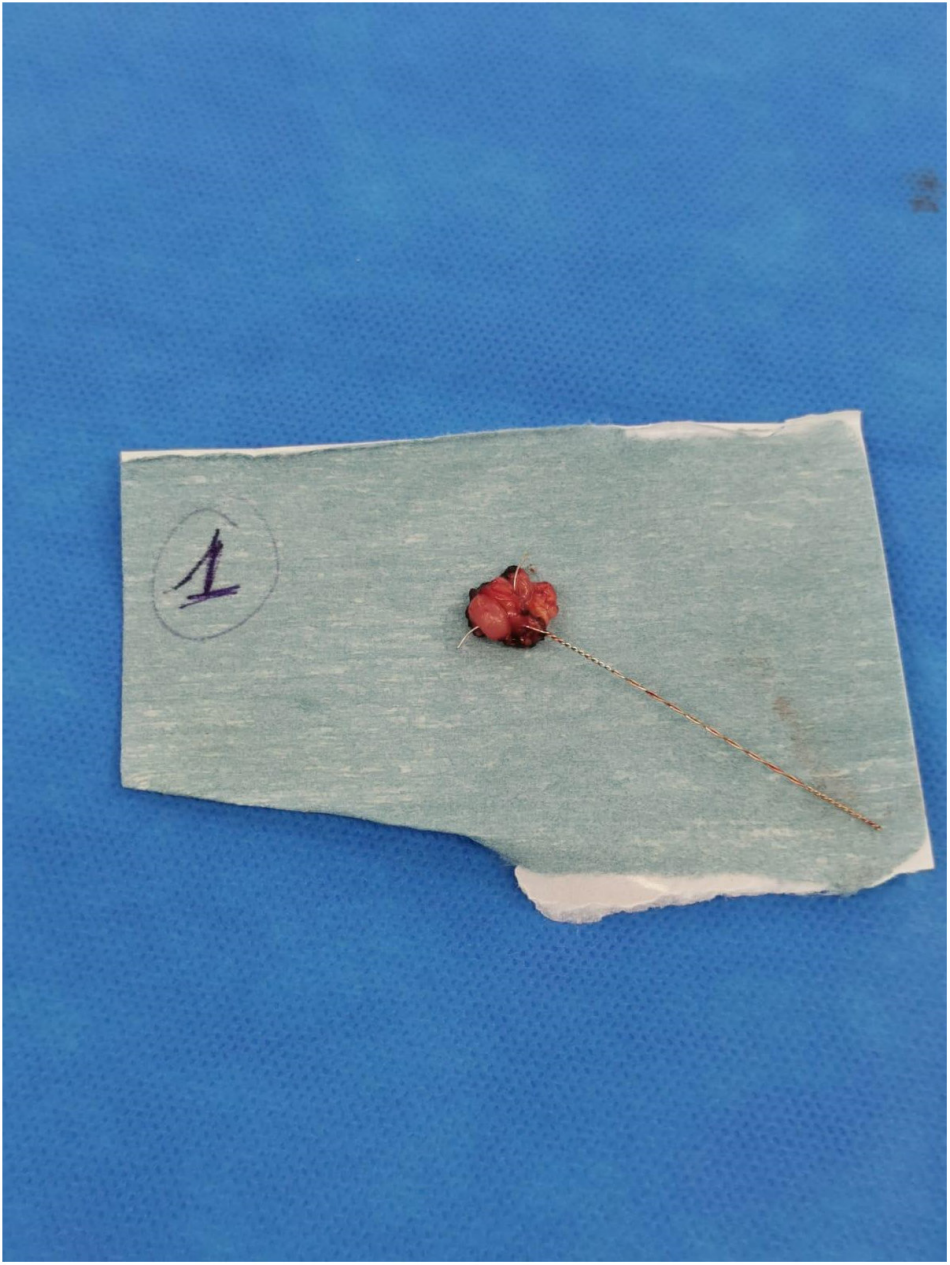
Adenectomy patch with hook‐wire in place.

Immediately after the insertion of the hook‐wire, the surgeon made a one‐centimeter incision close to the hook‐wire and then performed a dissection guided by the wire. (Figure 6). After its excision, the lesion specimen was sent for histopathological analysis (Figure 7). Patients did not receive postoperative antibiotic prophylaxis.

### 2.6 Data collection

The general characteristics of the patients were extracted from the medical records and listed in Table 3. Operating time was defined as the onset of GA to the end of surgery, that is, included the time of hookwire placement. This was recorded by two independent readers unaware of patient's group allocation and the other reader's findings. The data were retrieved from two data sources: Qbloc surgical process management software® (Evolucare) and the anesthesia records. In case of discrepancies in operating time between the two sources, the shorter time was selected for each of the two groups H+ and H−. Relevant data on intra‐ and postoperative complications and histological findings were retrieved from the clinical medical records.

**TABLE 3:** Comparison of the general characteristics of the two groups (H+ and H−).

Features	H+	H−	*p*	Total
Number of patients	9	17	–	26
Mean age (years)	63 ± 10	62 ± 16	0.571	–
Female gender	3 (33%)	6 (35%)	1	9
BMI	25.3 ± 4	27.6 ± 5.2	0.499	–
History of cancer	7 (78%)	10 (59%)	0.418	17
History of neck surgery	5 (56%)	6 (35%)	0.418	11
History of radiotherapy	5 (56%)	5 (29%)	0.234	10
Anticoagulant/antiaggregant use	1 (11%)	4 (24%)	0.628	5
Diabetes	0	3 (18%)	0.529	3
Location of adenopathy	–	–	0.4614	–
Lesion size (mm)	13.7 ± 5.4	18.6 ± 8.5	0.122	–
Cytopuncture performed	7 (78%)	9 (53%)	0.744	16
Median surgery lag time: days (IQ1; IQ3)	12 (9; 15)	10 (8; 21)	0.8600	–

Abbreviations: BMI, body mass index; H+, hook‐wire group; H−, nonhook‐wire group; IQ1; IQ3, interquartile range; mm, millimeter.

### 2.7 Statistical methodology

Statistical analyses were performed with SPSSV 25.0 software (IBM, USA, Chicago). Quantitative data were expressed as mean ± standard deviation or median (IQ1; IQ3) and qualitative data as percentage or numbers. The difference in quantitative data was evaluated by the Mann Whitney *U* test and the difference in qualitative data by the Fisher test (*p* < 0.05). A univariate analysis of the compared outcomes was done (significance set at *p* < 0.20). For outcomes representing a between‐group difference in univariate analysis, a further multivariate analysis was performed.

Cox regression model assessed the effect of hookwire on operative time (primary outcome of interest). The relationship between the hookwire (explanatory variable) and the operative time (dependent variable or primary outcome) was analyzed and adjusted for covariates (explanatory variables) or predictors (e.g., lesion size, radiotherapy history, neck surgical history, anticoagulant treatment).

A logistic regression assessed the effect of hookwire on postoperative complications (secondary outcomes). For hematoma, it was adjusted with anticoagulant treatment. For Infection, it was adjusted with immunodeficiency.

## 3 RESULTS

There was no statistically significant between group differences in patient characteristics (Table 3).

### 3.1 H+ group

This group included all patients (three women, six men; mean age: 62.8 ± 10 years) who had undergone adenectomy for suspicious nonpalpable cervical adenopathy using intraoperative US‐guided hook‐wire. One patient had two hook‐wire localizations at 7 months apart, one patient had four hook‐wires on the same day in three US‐detected suspicious lesions (two hook‐wires pierced the skin near the same target). One patient had a hook‐wire in contact with two adjacent suspicious lesions. In brief, 13 hook‐wire insertions were performed for localization of 12 nonpalpable suspicious lesions.

Surgical removal was performed under general anesthesia for all screened nodes, without intraoperative complications, with a median surgery lag time (i.e., duration between initial consultation and adenectomy) was 12 days (9; 15). The mean operative time, including the hook‐wire placement, was 26 ± 16 min. No patient had a bleeding or nerve damage. Postoperative complications are reported in Table 4.

**TABLE 4:** Assessment of operative time, histological result, and complications of adenectomy in the H+ group.

Patient	Location	Number of hook‐wire	Operating time (min)	Lesion size (mm)	Histology	Complication
1	IA left	1	15	15	SCC	Wound healing disorder
1	IA left	1	22	8	SCC	0
2	IV left	1	22	15	PTC	0
3	IIA right	1	55	11	SCC	0
4	IB right	1	20	11	SCC	0
5	IIA left	1	20	10	Benign	0
5	IIB left	1	10	10	SCC	0
5	III left	2	12	10	SCC	0
6	IIB left	1	50	15; 8	Benign	0
7	IV left	1	48	20	Necrosis	0
8	IB right	1	25	25	Benign	Wound healing disorder
9	IV right	1	13	20	Benign	0

Abbreviations: min, minutes; mm, millimeter; PTC, papillary thyroid carcinoma; SCC, squamous cell carcinoma.

Of note, one patient underwent two surgical interventions with nonconclusive biopsy results for suspicion of oral cavity floor hypermetabolism. The same patient showed recurrent disease when using US‐guided hookwire localization. Histological analysis was conclusive in 100% of cases (Table 3). The mean lesion size was 13.7 ± 5.4 mm.

### 3.2 H− group

Between January 2017 and January 2021, 17 patients (8 women, 11 men, mean age: 62 ± 16 years) underwent surgery under general anesthesia for suspicious nonpalpable cervical adenopathy without US‐guided hookwire localization. The mean operating time was 43 ± 22 min. Median surgery lag time was 10 days (8; 21). Postoperative complications are reported in Table 5.

**TABLE 5:** Assessment of operative time, histological diagnosis accuracy, and adverse events of adenectomy in the H− group.

Patient	Location	Operating time (min)	Lesion size (mm)	Histology	Adverse event
1	IV right	40	40	Sarcoidosis	0
2	IV left	55	6	Benign	0
3	IV right	30	28	Ovarian adenocarcinoma	0
4	III right	45	30	Lymphoma	0
5	IIA, III left	49	20	Lymphoma	0
6	IB right	115	11	SCC	0
7	IIA right	30	20	SCC	Hematoma
8	IB, IIA right	45	15	Breast adenocarcinoma and lymphoma	0
9	IB left	45	20	SCC	0
10	IIA left	20	17	PTC	Hematoma
11	IV left	20	12	Lymphoma	0
12	III left	35	15	Sarcoidosis	0
13	V right	30	25	Inflammatory	0
14	IIA right	36	–	–	Failure
15	IIA left	27	9	Benign	0
16	IIB right	70	20	EC	0
17	IIA left	40	9	Renal cell carcinoma	0

Histopathological diagnosis was accurate in 94% (*n* = 16). One diagnosis was not comprehensively done due to the failure of adenectomy (i.e., involvement of vascular structures). The mean lesion size was 18.6 ± 8.5 mm.

### 3.3 Comparison of outcomes and impact of hookwire

Between groups operative times of cervical adenectomies were significantly lower in H+, without any difference in diagnostic performance and adverse events rates (e.g., hematoma, infection) (Table 6). The hookwire use was significantly related with operative time according to patient's factors of past history of radiotherapy and neck surgery (Table 7).

**TABLE 6:** Comparison of operative time, histological diagnosis accuracy and adverse events of adenectomy between the two groups H+ and H−.

Group		H+	H−	*p*
Operating time in min (Mean standard ± deviation)		26 ± 16	43 ± 22	**0**.**020** **0**.**039** *
Histopathological diagnostic accuracy (*n*)		100% (12)	94% (16)	1
Lesion size (mm)		13.7 ± 5.4	18.6 ± 8.5	0.270
Adverse events	Wound healing disorder	2	0	0.162
	Hematoma	0	2	0.498
	Failure of adenectomy	0	1	1

*Note:* Bold values represent statistical significance.

* Multivariate analysis was performed after significant difference according to univariate analysis.

**TABLE 7:** Relationship between operative time and covariate of hookwire and adjusted for patient's factors.

	*p*	Odds ratio	95% CI	
			Lower	Upper
Hookwire	0.015	2.958	1.233	7.096
Size	0.890	1.004	0.949	1.062
Radiotherapy history	0.037	0.380	0.154	0.943
Neck surgery history	0.047	2.410	1.013	5.729

*Note*: Cox regression model results.

## 4 DISCUSSION

Perioperative US‐guided hook‐wire localization of nonpalpable cervical adenopathy allowed a significantly shorter mean operative time by 17 min (*p* = 0.02) compared to no hook‐wire surgery. Histopathology diagnosis was conclusive in all cases (*n* = 12). There was no statistically significant difference in intra‐ and postoperative adverse events rates between H+ and H−.

Our mean operative time using US‐hookwire localization of lateral nonpalpable cervical adenopathy was consistent (26 ± 16 vs. 30.2 ± 4.6 min) with the literature on breast cancer.^43^

The hookwire placement was done according to planned surgical approach at the discretion of the surgeon. The time required for the radiologist to place the hookwire depends on the following parameters: number and depth of nodes, patient anatomy and tissue status (previous surgery, radiotherapy).

Vasovagal syncope is reported to be the main hookwire placement complication (i.e., 10% rate in case of placement under mammography and local anesthesia).^20^ Our perioperative hook‐wire placement under general anesthesia could help at reducing most reported complications using local anesthesia, for example patient discomfort, vasovagal syncope, and hookwire secondary displacement.

Hematoma is also a possible complication of hookwire placement, occurring in about 4% of cases according to the study by Köhler et al.^20^ The postplacement bleeding results of our US‐guided hook wire localization using a 20G needle were comparable (0% vs. 0.1%) with the relevant literature using 16–20G needles.^44^ Moreover, optimal real‐time US‐imaging of the target seems promising in prevention of damage to adjacent tissue structures (i.e., reduction of adverse events).

There is a risk of tumor cell dissemination along the hookwire path similar to that reported in the track of needle biopsy. In cervical pathology, the risk of dissemination after biopsy sampling is low (for two cases (0.001%) of 1803) biopsies of cervical lesions, according to a large review using 18–22G needles.^45^

According to the literature on breast cancer, US‐guided hookwire localization has the following risk factors for failure: difficulty of placement in dense fibrous tissue, secondary displacement, unmovable placement, fragile wire, pneumothorax, patient discomfort, unsuitable specimen, discomfort during surgical approach or surgeon's injury.^17^, ^46^

Intraoperative US‐guided hookwire localization of nonpalpable cervical lesions is an effective technique but has several drawbacks. It requires radiologists trained in both cervical US and hookwire technique. The guide wire must perforate the skin as close as possible to the surgical incision and proceed along the shortest and most direct path towards the target. This requires a comprehensive collaboration between the surgeon and radiologist as early as preoperative scheduling for the intervention. In our study, all hook‐wires were placed in the operating room after GA. The other feasible option would be to schedule the hookwire placement and stabilization a few hours before surgery at the radiology unit.

Hartl et al. proposed the use of US‐guided charcoal tattooing,^12^ allowing 84% localization success of screened nodes. There was a 16% localization failure due to tattoo factors such as remote diffusion or dilution in a hematoma. The use of radioactive markers to facilitate the removal of nonpalpable cervical nodes (neoplasia) has been described in recurrent thyroid cancer. However, such tools diagnostic accuracy depends on the nodes status as well as the type of marker used. In the Travagli et al study, 26% of lymph node metastases were not detected by the probe intraoperatively.^14^ There is also some risk of radiation exposure to the surgeon and the patient. Such tools compared with US do not provide real‐time imaging, cost more and are not as readily feasible to use.^13^, ^14^, ^33^

Our study had several limitations such as its retrospective design and small sample size. The latter is larger than the reported relevant series on the use of hookwire.^27^ Our study was not designed to compare the superiority of US‐guided hookwire surgery of nonpalpable cervical lesions versus adenectomy. The strength of this study lies in its comprehensive collaboration between the surgeon and radiologist as early as preoperative scheduling for the intervention. Of note, the same team of surgeon—radiologist performed H− and H+ adenoectomies. All hook‐wires were placed in the operating room after GA.

We believe that our study opens the avenue to further prospective controlled trials. It conveys the usefulness of this tool for early diagnosis and management of nonpalpable cervical lesions and suspicions of recurrence. Early diagnosis of disease recurrences at an asymptomatic stage, can improve patients' outcome and overall survival.^11^, ^47^ Of note, in the absence of remote metastasis, lymph node status provides the mainstay prognostic factor in the management of ENT neoplasia.^48^ Altogether, US‐guided hookwire surgery of ENT neoplasia could help at disease spread reduction and better patient outcome. This confers the use of such tool for other ENT indications (e.g., benign nonpalpable thyroglossal tract cyst, branchial cyst).

## 5 CONCLUSION

This study conveys the effectiveness of US‐guided hookwire localization for early diagnosis and removal of nonpalpable cervical lesions. This helps not only at timely management of suspicions of recurrence but also at prevention of disease spread. Our study opens the avenue to larger prospective controlled trials.

## AUTHOR CONTRIBUTIONS

**William Bran:** Conceptualization (lead); data curation (lead); formal analysis (lead); investigation (lead); methodology (lead); supervision (lead); validation (lead); visualization (lead); writing – original draft (lead); writing – review and editing (lead). **Sonia Sahli‐vivicorsi:** Conceptualization (equal); data curation (equal); formal analysis (equal); investigation (equal); methodology (equal); supervision (equal); validation (equal); writing – original draft (equal); writing – review and editing (equal). **Romain Cadieu:** Conceptualization (equal); data curation (equal); formal analysis (equal); investigation (equal); methodology (equal); supervision (equal); validation (equal); visualization (equal); writing – original draft (equal); writing – review and editing (equal). **Zarrin Alavi:** Formal analysis (equal); project administration (equal); resources (equal); validation (equal); writing – original draft (equal); writing – review and editing (equal). **Jean‐Christophe Leclère:** Conceptualization (lead); data curation (lead); formal analysis (lead); investigation (lead); methodology (lead); supervision (lead); validation (lead); visualization (lead); writing – original draft (lead); writing – review and editing (lead).

## CONFLICT OF INTEREST STATEMENT

The authors have no conflict of interest to declare.

## Data Availability

All study data will be available upon request to the publication Corresponding Author Dr. William Bran: wfcbran@gmail.com.
